# A Proteomic View of Cellular Responses to Anticancer Quinoline-Copper Complexes

**DOI:** 10.3390/proteomes7020026

**Published:** 2019-06-24

**Authors:** Bastien Dalzon, Joanna Bons, Hélène Diemer, Véronique Collin-Faure, Caroline Marie-Desvergne, Muriel Dubosson, Sarah Cianferani, Christine Carapito, Thierry Rabilloud

**Affiliations:** 1 Chemistry and Biology of Metals, Univ. Grenoble Alpes, CNRS UMR5249, CEA, IRIG,CBM, F-38054 Grenoble, France; bastien.dalzon@gmail.com (B.D.); veronique.collin@cea.fr (V.C.-F.); 2 Laboratoire de Spectrométrie de Masse BioOrganique (LSMBO), Université de Strasbourg, CNRS, IPHC UMR 7178, 67000 Strasbourg, France; joanna.bons@etu.unistra.fr (J.B.); hdiemer@unistra.fr (H.D.); sarah.cianferani@unistra.fr (S.C.); ccarapito@unistra.fr (C.C.); 3 Nanosafety Platform, Medical Biology Laboratory (LBM), Univ. Grenoble-Alpes, CEA, 17 rue des Martyrs, F-38054 Grenoble, France; caroline.desvergne@cea.fr (C.M.-D.); muriel.dubosson@cea.fr (M.D.)

**Keywords:** anticancer copper complex, hydroxyquinoline copper complex, leukemic cells, proteomics, two-dimensional electrophoresis, proteasome, glutathione, actin cytoskeleton, antioxidant defense

## Abstract

Metal-containing drugs have long been used in anticancer therapies. The mechansims of action of platinum-based drugs are now well-understood, which cannot be said of drugs containing other metals, such as gold or copper. To gain further insights into such mechanisms, we used a classical proteomic approach based on two-dimensional elelctrophoresis to investigate the mechanisms of action of a hydroxyquinoline-copper complex, which shows promising anticancer activities, using the leukemic cell line RAW264.7 as the biological target. Pathway analysis of the modulated proteins highlighted changes in the ubiquitin/proteasome pathway, the mitochondrion, the cell adhesion-cytoskeleton pathway, and carbon metabolism or oxido-reduction. In line with these prteomic-derived hypotheses, targeted validation experiments showed that the hydroxyquinoline-copper complex induces a massive reduction in free glutathione and a strong alteration in the actin cytoskeleton, suggesting a multi-target action of the hydroxyquinoline-copper complex on cancer cells.

## 1. Introduction

Metal-containing drugs, i.e., metal-organic complexes, also known as metallodrugs, have been used to combat cancer for quite a long time. The oldest metal-based drugs in this field are clearly platinum complexes [1], which act mainly by inducing severe DNA damage [2,3]. Because of the side effects of these drugs and of the emergence of resistances, other metal complexes have been investigated as anticancer agents. Various metals such as gold [4,5], copper [6,7], zinc [8] or ruthenium [9,10,11] have been investigated (reviewed in [12]). Various mechanisms of actions have been proposed for these non-platinum metallodrugs. For ruthenium complexes, their binding to proteins have been extensively studied, they suggest that different ruthenium metallodrugs bind to (and may inhibit) different proteins [13]. 

Taking gold complexes as another example, the suggested mechanisms range from DNA lesions [14] to mitochondrial stress [15] and to proteasome inhibition [4]. The facts that proteasome inhibition is a well-established avenue in anticancer treatments (reviewed in [16]), and that metals are known inhibitors of the proteasome [17,18,19], have paved the way to thorough investigations of this effect in metallodrugs [4,6,17,20,21,22,23,24,25,26,27]. Nevertheless, other mechanisms have been put forward to explain the anticancer activities of the metallodrugs, including direct induction of apoptosis [15], topoisomerase inhibition [28] or redox imbalance [29,30]. 

One of the attractive features of the metallodrugs proposed to date is that they often rely on organic moieties that are established drugs by themselves in other medical fields, so that their toxicology is already well known. As an example, disulfiram, which has been proposed against cancer as a copper [31] or zinc [8] complex is also well known for treatment against alcoholism [32]. Clioquinol, a copper complex which has also been proposed against cancer [20,21,25], was first used alone as an antiparasitic drug [33,34]. 

Besides these simple metal-drugs complexes, other research avenues in metallodrugs have focused on more elaborate chemistries, in which a metal binding moiety is covalently grafted onto drug-related organic molecules. The use of ferrocene as the metal component, grafted onto various prodrugs, has been proposed in several approaches [35,36,37]. In addition to iron, gold and silver metal carbene complexes have been proposed as metallodrugs [38].

Knowing as much as possible about the actions of these metallodrugs on cancer cells is not just an academic exercise; it may impact their future use as drugs in clinics, which is still in progress [39]. For example, antioxidant capacity has been proposed as a mechanism for cancer cells to resist to proteasome inhibitors [40]. Thus, if a metallodrug is able to inhibit proteasome and antioxidant defenses at the same time, it may overcome classical cell resistances. Furthermore, it has been demonstrated on ferrocene-based metallodrugs that different organic moieties coupled to the same metal-containing moiety lead to different biological consequences [41].

When multiple mechanisms of action of a chemical on cells are suspected, omics approaches are very relevant and interesting, as they do not need pre-conceived hypotheses other than the homeostatic principles [42]. It is therefore not surprising that such approaches have been used to increase our knowledge on the mechanisms of actions of anticancer metallodrugs, as exemplified by work carried out on ruthenium [43] and gold complexes [44], leading to the discovery of new modes of action for these chemicals. 

We therefore decided to use a proteomic approach to further investigate the effects of copper complexes on leukemic cells, as they are attractive therapeutical targets [45]. As a cellular model, we have used the RAW 264.7 cell line, which is a myeloid leukemic mouse cell line. As the copper complex, we have used a complex of copper with 8-hydroxyquinoline. This organic compound shows an interesting pharmacology [46], including a noticeable anticancer activity on cells [6,24], and is the parent compound for several metallo-prodrugs currently under investigation [23,26,29,47] including the classical drug clioquinol [20,21,39]. Furthermore, copper is a very attractive metal to be investigated, as it appears to be more toxic on cancer cells than on normal cells, provided that its concentration can be kept high enough in cells [48,49].

## 2. Materials and Methods 

Unless specified otherwise, the chemicals used in this work were purchased from Sigma-Millipore and were at least 99% pure. As a copper source for the copper complexes, we used a titrated copper sulfate solution (4% catalog number C2284).

### 2.1. Cell Culture

The mouse monocyte/macrophage cell line RAW264.7 was purchased from the European Cell Culture Collection (Salisbury, UK). The cells were cultured in RPMI 1640 medium + 10% fetal bovine serum. Cells were seeded every two days at 200,000 cells/mL and harvested at 1,000,000 cells per ml. For treatment with chemicals, the following scheme was used: cells were first seeded at 500,000 cells/mL in T175 flasks (50 mL per flask). They were exposed to 8-hydroxyquinoline or to the 8-hydroxyquinoline-copper complex (2:1) on the following day and harvested after a further 24 h in culture. Cell viability was measured by the neutral red uptake assay [50]. All experiments were carried out at least in triplicate on independent cultures. 

### 2.2. Ion Quantification by ICP-MS

To measure ion uptake in cells, the cells were harvested after exposure to 8-hydroxyquinoline or to the 8-hydroxyquinoline-copper complex by scraping in buffer A (Hepes 50 mM pH 7.5, sorbitol 200 mM, magnesium acetate 2 mM), and collected by centrifugation (500 g, 5 min). After a further rinse in buffer A, the cells were lysed by incubation in 10 cell pellet volume of lysis buffer (Hepes 50 mM pH 7.5, magnesium acetate 2 mM, tetradecyldimethylammonio propane sulfonate (SB 3-14) 0.15% (*w*/*v*)) for 20 min on ice, and the protein concentration in the extracts, determined by a modified dye-binding assay [51]. 

The extracts were mineralized by the addition of three volumes of suprapure 65% HNO_3_ per volume of extract and incubation on a rotating wheel at room temperature for 18 h. 

Mineralized samples were then diluted in ultrapure grade HNO_3_ (1% (*v*/*v*)) and analyzed on a Nexion 300X ICP-MS (Perkin Elmer, Waltham, MA, USA) equipped with a concentric nebulizer and operated in standard mode. External calibration was performed using certified ionic Cu and Zn ICP-MS standards and Yttrium was used as an internal standard. 

The technique was validated based on linearity, repeatability, reproducibility, accuracy, inter-sample contamination criterions, and the analytical limit of detection (LoD) determined as the sum of the mean and three standard deviations of 20 ultrapure water samples. For this experiment, the LoD was 0.0017 µg/L for Cu and 0.022 µg/L for Zn. 

### 2.3. Proteomics

The 2D gel based proteomic experiments were essentially carried out as previously described [52], on independent biological triplicates. However, detailed material and methods are provided for the sake of consistency. 

#### 2.3.1. Sample Preparation

The cells were collected by scraping, and then washed three times in PBS. The cells were then washed once in TSE buffer (Tris-HCl 10 mM pH 7.5, sucrose 0.25 M, EDTA 1 mM), and the volume of the cell pellet was estimated. The pellet was resuspended in its own volume of TSE buffer. Then 4 volumes (respective to the cell suspension just prepared) of concentrated lysis buffer (urea 8.75 M, thiourea 2.5 M, CHAPS 5% (*w*/*v*), TCEP-HCl 6.25 mM, spermine base 12.5 mM) were added and the solution was let to extract at room temperature for 1 h. The nucleic acids were then pelleted by ultracentrifugation (270,000 g at room temperature for 1 h), and the protein concentration in the supernatant was determined by a modified dye-binding assay [43]. Carrier ampholytes (Pharmalytes pH 3–10) were added to a final concentration of 0.4% (*w*/*v*), and the samples were kept frozen at −20 °C until use.

#### 2.3.2. Isoelectric Focusing 

Home-made 160 mm long 4–8 linear pH gradient gels [53] were cast according to published procedures [54]. Four mm-wide strips were cut, and rehydrated overnight with the sample, diluted in a final volume of 0.6 mL of rehydration solution (urea 7 M, thiourea 2 M, CHAPS 4%, carrier ampholytes 0.4% (Pharmalytes 3–10) and dithiodiethanol 100 mM [55].

The strips were then placed in a Multiphor plate (GE Healthcare), and IEF was carried out with the following electrical parameters: 100V for 1 h, then 300 V for 3 h, then 1000 V for 1 h, then 3400 V up to 60–70 kVh. After IEF, the gels were equilibrated for 20 min in Tris 125mM, HCl 100mM, SDS 2.5%, glycerol 30% and urea 6 M [56]. They were then transferred on top of the SDS gels and sealed in place with 1% agarose dissolved in Tris 125 mM, HCl 100 mM, SDS 0.4% and 0.005% (*w*/*v*) bromophenol blue. 

#### 2.3.3. SDS Electrophoresis and Protein Detection

Ten percent gels (160 × 200 × 1.5 mm) were used for protein separation. The Tris taurine buffer system [57] was used and operated at a ionic strength of 0.1 and a pH of 7.9. The final gel composition is thus Tris 180 mM, HCl 100 mM, acrylamide 10% (*w*/*v*), bisacrylamide 0.27%. The upper electrode buffer is Tris 50 mM, Taurine 200 mM, SDS 0.1%. The lower electrode buffer is Tris 50 mM, glycine 200 mM, SDS 0.1%. The gels were run at 25V for 1hour, then 12.5 W per gel until the dye front has reached the bottom of the gel. Detection was carried out by a tetrathionate silver staining [58]. 

#### 2.3.4. Image Analysis

The gels were scanned after silver staining on a flatbed scanner (Epson perfection V750), using a 16 bits grayscale image acquisition. The gel images were then analyzed using the Delta 2D software (v 4.7). Spots that were never expressed above 100 ppm of the total spots were first filtered out. Then, significantly-varying spots were selected on the basis of their Student T-test p-value between the treated and the control groups. Spots showing a p-value lower than 0.05 were selected. 

The false discovery rate was controlled by a combination of approaches, including the classical Benjamini-Hochberg FDR [59] or the more recent Sequential Goodness of Fit approaches [60,61]. Global analysis of the proteomic results was carried out by the PAST statistical software [62]. 

#### 2.3.5. Mass Spectrometry

The spots selected for identification were excised from silver-stained gels and destained with ferricyanide/thiosulfate on the same day as silver staining in order to improve the efficiency of the identification process [63]. In gel digestion was performed with an automated protein digestion system, MassPrep Station (Waters, Milford, MA, USA). The gel plugs were washed twice with 50 µL of ammonium hydrogen carbonate (NH_4_HCO_3_) 25 mM and 50 µL of acetonitrile. The cysteine residues were reduced by 50 µL of 10 mM dithiothreitol at 57 °C and alkylated by 50 µL of iodoacetamide 55 mM. After dehydration with acetonitrile, the proteins were cleaved in gel with 10 µL of 6.5 ng/µL of modified porcine trypsin (Promega, Madison, WI, USA) in NH_4_HCO_3_ 25 mM. The digestion was performed overnight at room temperature. The generated peptides were extracted with 30 µL of 60% acetonitrile in 0.1% formic acid. Acetonitrile was evaporated under vacuum before nanoLC-MS/MS analysis.

NanoLC-MS/MS analysis was performed using a nanoACQUITY Ultra-Performance-LC (Waters Corporation, Milford, MA, USA) coupled to the Synapt^TM^ High Definition Mass Spectrometer^TM^ (Waters Corporation, Milford, MA, USA), or to the TripleTOF 5600 (Sciex,Framingham, MS, USA). 

The nanoLC system was composed of ACQUITY UPLC^®^ CSH130 C18 column (250 mm × 75 µm with a 1.7 µm particle size, Waters Corporation, Milford, MA, USA) and a Symmetry C18 precolumn (20 mm × 180 µm with a 5 µm particle size, Waters Corporation, Milford, MA, USA). The solvent system consisted of 0.1% formic acid in water (solvent A) and 0.1% formic acid in acetonitrile (solvent B). 4 µL of sample were loaded into the enrichment column during 3 min at 5 µL/min with 99% of solvent A and 1% of solvent B. Elution of the peptides was performed at a flow rate of 300 nL/min with a 8–35% linear gradient of solvent B in 9 min. 

The Synapt^TM^ High Definition Mass Spectrometer^TM^ (Waters Corporation, Milford, MA, USA) was equipped with a Z-spray ion source and a lock mass system. The system was fully controlled by MassLynx 4.1 SCN639 (Waters Corporation, Milford, MA, USA). The capillary voltage was set at 2.8 kV and the cone voltage at 35 V. Mass calibration of the TOF was achieved using fragment ions from Glu-fibrino-peptide B on the [50;2000] *m/z* range. Online correction of this calibration was performed with Glu-fibrino-peptide B as the lock-mass. The ion [M + 2H]^2+^ at *m/z* 785.8426 was used to calibrate MS data and the fragment ion [M + H]^+^ at *m/z* 684.3469 was used to calibrate MS/MS data during the analysis.

For tandem MS experiments, the system was operated with automatic switching between MS (0.5 s/scan on *m/z* range [150;1700]) and MS/MS modes (0.5 s/scan on *m/z* range [50;2000]). The two most abundant peptides (intensity threshold 20 counts/s), preferably doubly and triply charged ions, were selected on each MS spectrum for further isolation and CID fragmentation using collision energy profile. Fragmentation was performed using argon as the collision gas. 

Mass data collected during analysis were processed and converted into pkl files using ProteinLynx Global Server 2.3 (Waters Corporation, Milford, MA, USA). Normal background subtraction type was used for both MS and MS/MS with 5% threshold and polynomial correction of order 5. Smoothing was performed on MS/MS spectra (Savitsky-Golay, 2 iterations, window of 3 channels). Deisotoping was applied for MS (medium deisotoping) and for MS/MS (fast deisotoping).

The TripleTOF 5600 was operated in positive mode, with the following settings: ionspray voltage floating (ISVF) 2300 V, curtain gas (CUR) 10, interface heater temperature (IHT) 150, ion source gas 1 (GS1) 2, declustering potential (DP) 80 V. Information-dependent acquisition (IDA) mode was used with Top 10 MS/MS scans. The MS scan had an accumulation time of 250 ms on *m/z* [400;1250] range and the MS/MS scans 100 ms on *m/z* [150;1800] range in high sensitivity mode. Switching criteria were set to ions with charge state of 2–4 and an abundance threshold of more than 500 counts, exclusion time was set at 4 s. IDA rolling collision energy script was used for automatically adapting the CE. Mass calibration of the analyser was achieved using peptides from digested BSA. The complete system was fully controlled by AnalystTF 1.7 (Sciex) Raw data collected were processed and converted with MSDataConverter in mgf peak list format.

For protein identification, MS/MS data were interpreted using a local Mascot server with MASCOT 2.5.1 algorithm (Matrix Science, London, UK) against UniProtKB/SwissProt (version 2018_11, 558,898 sequences), without taxonomical restrictions. Spectra were searched with a mass tolerance of 15 ppm for MS and 0.05 Da for MS/MS data, allowing a maximum of one trypsin missed cleavage. Carbamidomethylation of cysteine residues and oxidation of methionine residues were specified as variable modifications. Protein identifications were validated with at least two peptides with Mascot ion score above 30.

Classical contaminants from human skin (keratins, filaggrin, desmoglein, involucrin) were removed from identifications. To cope with multiple identification issues from single 2D gel spots, univocal identifications were reported when the fist candidate was identified by at least twice more unique peptides than the next candidate or represented at least twice more spectra than the next candidate, and corresponded to the correct species (*Mus musculus*). This allowed removal of contaminating serum proteins (*Bos taurus*) from the identifications. In order to decrease the severity of the multiple identification problem, a graded identification strategy was used. A first identification of the spots of interest was attempted on analytical gels (loaded with 150µg protein, silver stained) when unsuccessful, more heavily loaded gels were used (250 µg protein, silver stained). If still unsuccessful, micropreparative gels (1 mg protein, colloidal Coomassie Blue-stained) were used. This strategy limited the problems associated with increased diffusion and streaking of proteins on 2D gels at high protein loads [64]. 

### 2.4. Phagocytosis and Particle Internalization Assay

The phagocytic activity was measured using fluorescent latex beads (1 µm diameter, green labelled, catalog number L4655 from Sigma, St Louis, MO, USA), as described previously [65]. After a 2h30 treatment with the fluorescent beads in culture medium at 37 °C, the cells were harvested in PBS). Phagocytic activity was measured by flow cytometry on a FacsCalibur instrument (Beckton Dickinson, Franklin Lakes, NJ, USA). 

### 2.5. F-Actin Staining

The experiments were performed essentially as previously described [66]. The cells were cultured on coverslips placed in 6-well plates, and exposed to 8-hydroxyquinoline or to the 8-hydroxyquinoline-copper complex as described above. At the end of the exposure time, cells were washed twice for 5 min at 4 °C in PBS, fixed in 4% paraformaldehyde for 30 min at room temperature. After two washes (5 min/4 °C in PBS), they were permeabilized in 0.1% Triton X100 for 5 min at room temperature. After two more washes in PBS, Phalloidin-Atto 550 (Sigma (500 nM) was added to the cells and let for 20 min at room temperature in the dark. Coverslips-attached cells were washed, placed on microscope slides (Thermo Scientific, Waltham, MS, USA) using a Vectashield mounting medium containing DAPI (Eurobio, Paris, France) and imaged using a Zeiss LSM 800 confocal microscope (Zeiss, Jena, Germany). The images were processed using the ImageJ software.

### 2.6. Mitochondrial Transmembrane Potential Measurement

The mitochondrial transmembrane potential was assessed by Rhodamine 123 uptake [52], using a low Rhodamine concentration (80 nM) to avoid intramitochondrial fluorescence quenching that would result in a poor estimation of the mitochondrial potential [67]. The samples were analyzed by flow cytometry on a FacsCalibur instrument (Beckton Dickinson). Both the proportion of rhodamine positive cells and the mean fluorescence of this population were recorded in the analysis. 

### 2.7. Glutathione Assay

Classical glutathione assays are difficult to apply to macrophage-like cells, as the oxidizing activities present in their phagolysosomes oxidize the cytosolic reduced glutathione when the cells are homogenized. To alleviate this problem, we used a spectrophotometric assay utilizing the cellular glutathione S-transferases and the chlorodinitrobenzene substrate [68]. After exposure to 8-hydroxyquinoline or to the 8-hydroxyquinoline-copper complex in 6 well plates, cells were treated for 30 min with chlorodinitrobenzene (25 µM, final concentration in the culture medium), resulting in the conjugation of glutathione to the substrate and consumption of free reduced glutathione [69,70]. The culture medium was removed and the cell layer was rinsed twice in Hepes NaOH buffer (10 mM pH 7.5) containing 2 mM magnesium acetate and 0.25 M sucrose. The cells were then lysed on a rocking table for 15 min in 800 µl of Hepes NaOH buffer (10 mM pH 7.5) containing 2 mM magnesium acetate and 1 mg/mL tetradecyldimethylammonio propane sulfonate (SB 3–14). The suspension was collected and centrifuged at 15,000 *g* for 15 min to pellet particulate material. The concentration of glutathione was determined by measuring the absorbance at 340 nm, using an extinction coefficient of 9600 M^−1^·cm^−1^ [68]. Results were normalized to the protein concentration of the cell extracts, determined by a modified dye-binding assay [51]. The final results were expressed in nmoles glutathione/mg protein.

### 2.8. NO Production 

The cells were grown to confluence in a 6 well plate and pre-treated with 8-hydroxyquinoline or to the 8-hydroxyquinoline-copper complex as described above. For the final 18 h of culture, half of the wells were treated with 100 ng/mL LPS (from salmonella, purchased from Sigma), and arginine monohydrochloride was added to all the wells (5 mM final concentration) to secure a high concentration of substrate for the nitric oxide synthase. After 18 h of incubation, the cell culture medium was recovered, centrifuged at 10,000 *g* for 10 min to remove cells and debris, and the nitrite concentration in the supernatants was read at 540 nm after addition of an equal volume of Griess reagent and incubation at room temperature for 30 min.

## 3. Results

**Figure 1:**
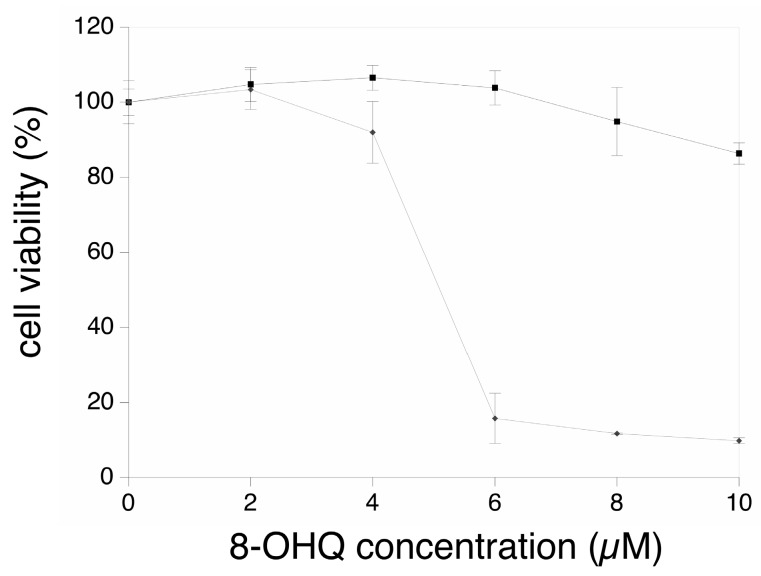
Dose-response viability curve.

We first investigated the working dose for the hydroxyquinoline-copper complex. To this purpose, cells were treated for 24 h with various doses of the copper complex, and with the corresponding doses of hydroxyquinoline. The results, shown on Figure 1, indicated a very strong decrease of viability at a complex concentration corresponding to 6 µM hydroxyquinoline and 3 µM copper. This led us to carry out all experiments with 4 µM hydroxyquinoline ± 2 µM copper. 

The cells were treated for 24 h with either 8-hydroxyquinoline alone (black curve, square symbols) or with the 8-hydroxyquinoline-copper 2:1 complex (grey curve, diamond symbols). The concentrations on the abscissae are expressed in µM 8-hydroxyquinoline.

As hydroxyquinoline and its derivatives have been shown to behave as ionophores for copper and zinc [71,72,73], we analyzed the intracellular concentration of these two metals after treatment with either hydroxyquinoline alone or with the hydroxyquinoline-copper complex. The results, shown in Table 1, indicated that the intracellular zinc concentration was constant in all conditions, and that treatment with hydroxyquinoline alone did not induce an influx of copper, while treatment with the copper complex resulted into a massive increase in intracellular copper concentration. 

### 3.1. Global Analysis of the Proteomic Results

**Figure 2:**
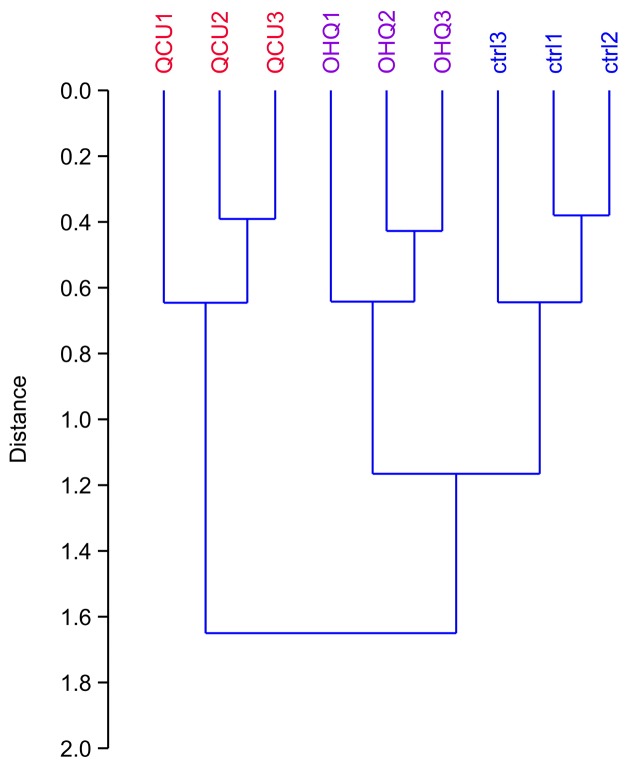
Global analysis of the proteomic experiment by hierarchical clustering. All the spots that showed a p-value <0.25 in either the hydroxyquinoline vs. control or the copper complex vs. control comparisons in the proteomic experiments were used for the calculation of the clustering tree. The PAST software package was used for the calculations, using a Euclidean distance and the Ward’s algorithm. This tree indicates the similarity between the various experimental groups (the higher the distance of the branching point between groups, the more dissimilar they are). Ctrl: unexposed cells. OHQ: cells exposed to 4 µM 8-hydroxyquinoline. QCu: cells exposed to 4 µM 8-hydroxyquinoline +2 µM copper sulfate. 3.2. Detailed Analysis of the Proteomic Results.

We then performed a proteomic screen using two-dimensional electrophoresis. The raw gel images are provided in Supplementary Figures S1–S3. Upon computerized analysis, 2444 spots were detected across the gel series, with a median CV of 21% (control), 20% (copper complex) and 16% (hydroxyquinoline alone). As a first test, we performed a global analysis of the results. To this purpose, we selected all spots showing a p-value < 0.25 by a Student T-test analysis in at least one of the two comparisons: copper complex vs. control or hydroxyquinoline vs. control. This allowed to decrease the noise provided by proteins with random variability, while selecting more than 50% of the spots (1426 out of 2444). This spot list was then analyzed by hierarchical clustering, and the results are shown on Figure 2. They indicated first that the within-group variability was much lower than the inter-group variability, which validated the overall quality of the proteomic analysis. It also indicated that the copper complex-treated group was separated from the other two groups, showing that the copper complex had a much stronger effect on the proteome than hydroxyquinoline alone. 

**Figure 3:**
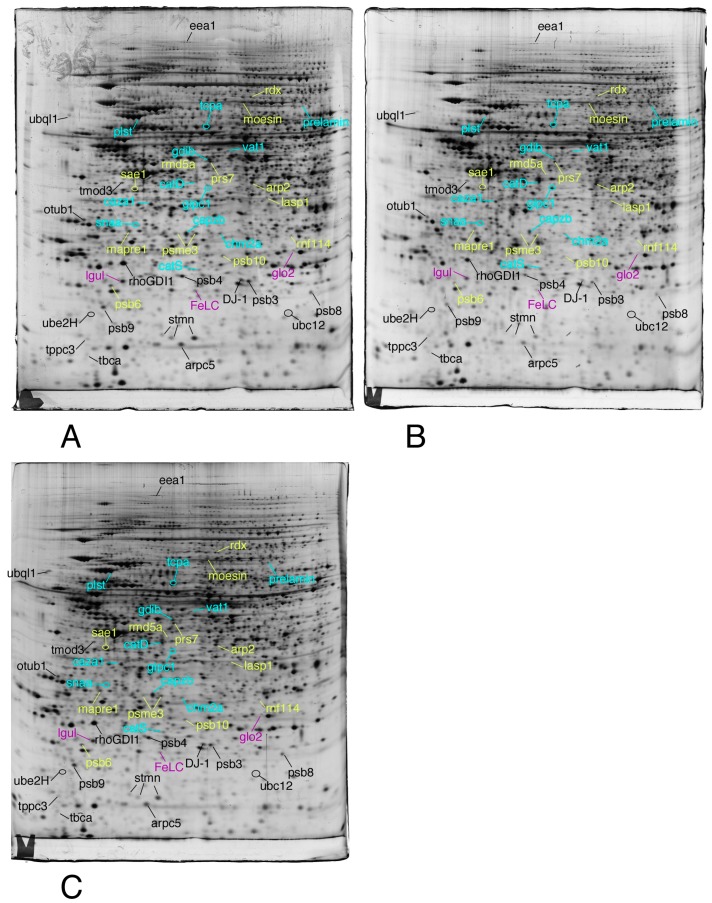
Proteomic analysis of total cell extracts by 2D electrophoresis. (**A**): gel obtained from unexposed cells; (**B**): gel obtained from cells exposed to 4 µM 8-hydroxyquinoline + 2µM copper sulfate; (**C**): gel obtained from cells exposed to 4 µM 8-hydroxyquinoline.

**Figure 4:**
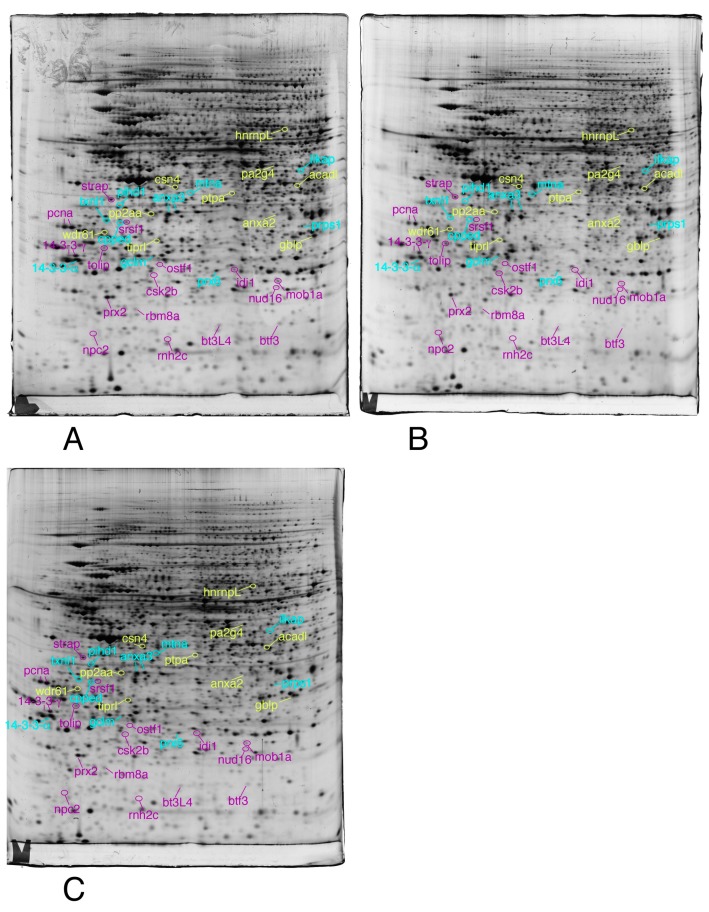
Proteomic analysis of total cell extracts by 2D electrophoresis. (**A**): gel obtained from unexposed cells; (**B**): gel obtained from cells exposed to 4 µM 8-hydroxyquinoline + 2 µM copper sulfate; (**C**): gel obtained from cells exposed to 4 µM 8-hydroxyquinoline.

**Figure 5:**
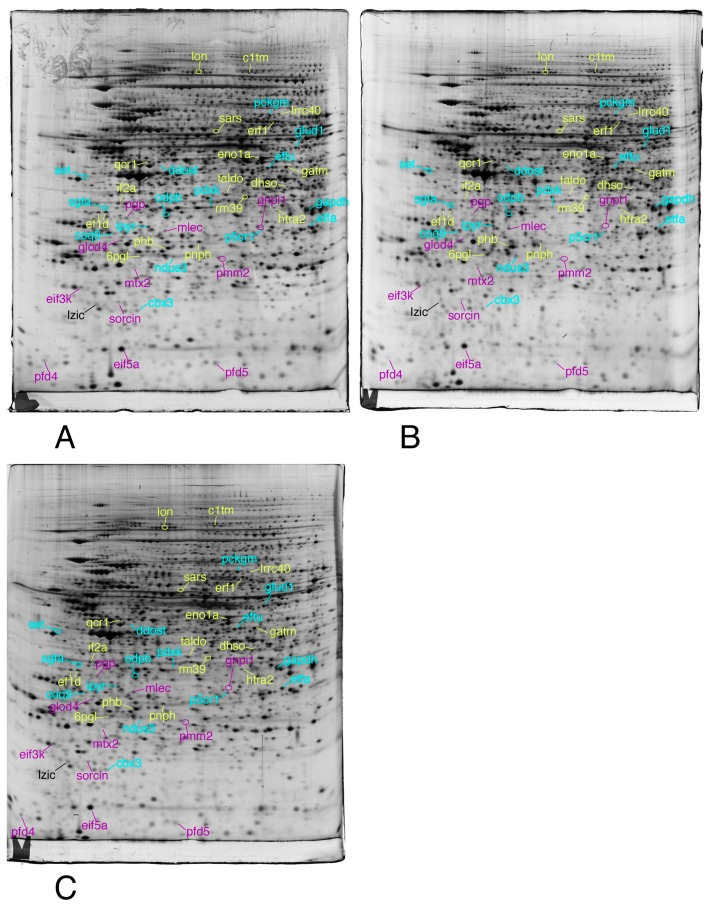
Proteomic analysis of total cell extracts by 2D electrophoresis. (**A**): gel obtained from unexposed cells; (**B**): gel obtained from cells exposed to 4 µM 8-hydroxyquinoline +2 µM copper sulfate; (**C**): gel obtained from cells exposed to 4 µM 8-hydroxyquinoline.

We then selected the spots modulated by at least one of the treatments, on the strict basis of the statistical tests, without any fold change threshold. This allowed to take into account coordinated but low amplitude changes that can be highly significant, as shown before [74]. The proteins were then identified by mass spectrometry. The results are shown on Figure 3, Figure 4 and Figure 5, the raw images, quantitative data and identification data being supplied in Supplementary Figures S1–S3, Supplementary Tables S1 and S2, respectively. 

Total cell extracts of RAW274.7 cells were separated by two-dimensional gel electrophoresis. The first dimensions covered a 4–8 pH range and the second dimension a 15–200 kDa range. Total cellular proteins (150 µg) were loaded on the first dimension gel. 

Only the proteins involved in the proteasome/ubiquitin pathway, cytoskeleton, homeostasis and vesicles/lysosomes are shown in this figure.

The same conditions as described in Figure 3 were applied to this figure 

Only the proteins involved in the RNA and nucleotide metabolism, lipid metabolism, redox control, and signaling are shown in this figure

The same conditions as described in Figure 3 were applied to this figure 

The proteins involved in mitochondria, central metabolism, protein production, as well as unclassified proteins are shown in this figure

In order to take into account the multiple testing issue, we submitted the modulated protein lists to statistical tests as the Benjamini-Hochberg FDR test [59] and the sequential goodness of fit tests [60,61]. The results, shown in Supplementary Tables S3 and S4, indicated that most of the proteins selected on the basis of the T-test also had a low FDR value with all the tested algorithms, validating their selection for further functional analysis.

As a first step, we analyzed the lists of modulated proteins by the DAVID pathway analysis tool. The results, shown in Supplementary Tables S5 and S6, indicated strongly modulated pathways, such as the ubiquitin/proteasome pathway, the mitochondrion, the cell adhesion-cytoskeleton pathway, carbon metabolism or oxido-reduction. As could be expected from the global analysis, more pathways were modulated upon treatment with the copper complex than with hydroxyquinoline alone. 

### 3.2. Targeted Validation Experiments

Even though the statistical tests indicated a strong robustness of the proteomic analysis, we decided to validate the proteomic results by targeted analyses. Based on the pathway analysis, we first measured the free glutathione concentration in cells, and obtained the following values: 

Control cells: 16.68 ± 1.52 nmoles/mg protein (range 15.15–18.02)

Cells treated with hydroquinoline alone: 16.22 ± 3.50 nmoles/mg protein (range 11.32–18.18)

Cells treated with the copper complex: 2.59 ± 3.37 nmoles/mg protein (range 0.89–7.66)

This very strong decrease upon treatment with the copper complex was statistically significant (*p* < 0.05 in Mann Whitney U test, *p* < 0.01 in Student T-test).

**Figure 6:**
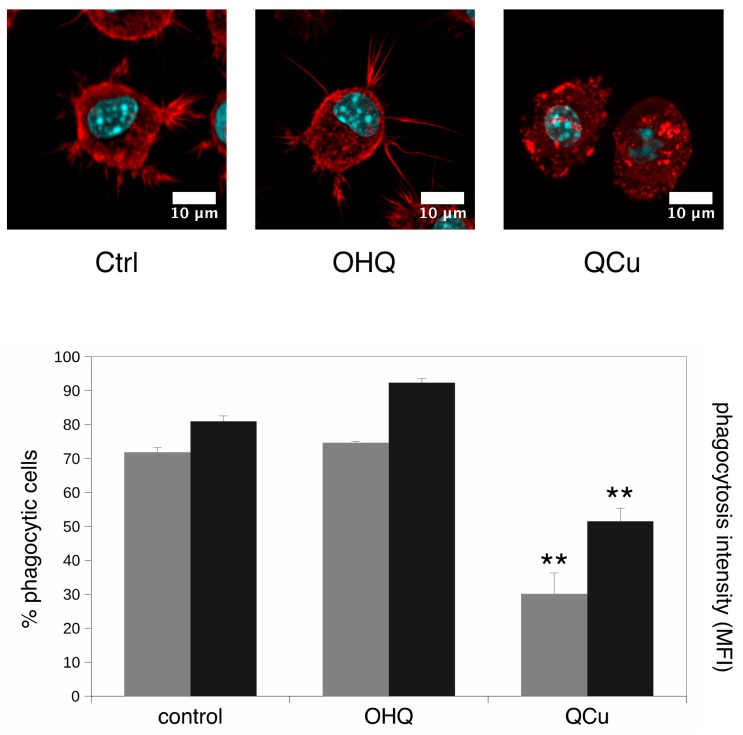
Actin cytoskeleton and phagocytosis. In the top panels, the actin cytoskeleton was visualized with fluorescent phalloidin and confocal microscopy. Only one confocal plane inside the cells is shown (going through the cell nucleus). Ctrl: unexposed cells. OHQ: cells exposed to 4 µM 8-hydroxyquinoline. Qcu: cells exposed to 4 µM 8-hydroxyquinoline +2 µM copper sulfate. In the bottom panel, the phagocytic capacity was assessed by fluorescent latex beads internalization. grey bars: proportion of positive cells in the viable cell population. black bars: mean cellular fluorescence. Ctrl: unexposed cells. OHQ: cells exposed to 4 µM 8-hydroxyquinoline. QCu: cells exposed to 4 µM 8-hydroxyquinoline +2 µM copper sulfate. Symbols indicate the statistical significance (Student *T*-test): **: *p* < 0.01.

As the adhesion-cytoskeleton and the lysosome pathways were also highlighted in the pathway analysis we also investigated the phagocytic activity, as it is dependent on both the integrity of the lysosomal pathway and on the actin cytoskeleton. We also studied the actin cytoskeleton by confocal microscopy. The results, shown on Figure 6, indicated once again no effect of hydroquinoline alone, but a strong effect of the copper complex, with the disappearance of pseudopodia and a strong decrease in the phagocytic activity, both in the proportion of phagocytic cells and in the number of beads internalized per cell. 

**Figure 7:**
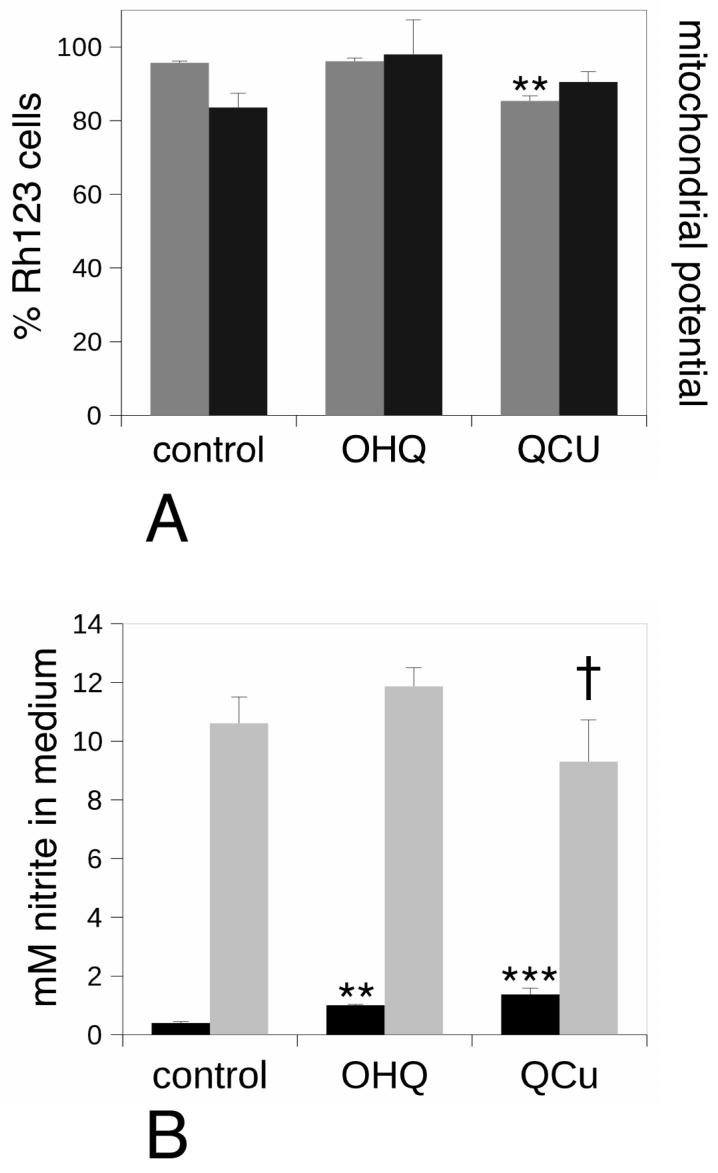
Analysis of the mitochondrial transmembrane potential and of the nitric oxide production. panel (**A**), the mitochondrial potential was analyzed by the rhodamine 123 accumulation method. Grey bars: proportion of positive cells in the viable cell population. Black bars: mean cellular fluorescence; Ctrl: unexposed cells. OHQ: cells exposed to 4 µM 8-hydroxyquinoline. QCu: cells exposed to 4 µM 8-hydroxyquinoline +2 µM copper sulfate. Symbols indicate the statistical significance (Student *T*-test): **: *p* < 0.01. In panel (**B**), the production of nitric oxide was analyzed. Black bars: production after treatment for 24 h with 4µM 8-hydroxyquinoline ± 2 µM copper sulfate. Grey bars: production after treatment for 24 h with 4 µM 8-hydroxyquinoline ±2 µM copper sulfate and stimulation with lipopolysaccharide for the last 18 h of culture. Ctrl: unexposed cells. OHQ: cells exposed to 4 µM 8-hydroxyquinoline. QCu: cells exposed to 4 µM 8-hydroxyquinoline + 2 µM copper sulfate. Symbols indicate the statistical significance (Student *T*-test): † *p* < 0.1; **: *p* < 0.01; ***: *p* < 0.001.

On the basis of the pathway analysis, we also investigated the mitochondrial activity by testing the mitochondrial transmembrane potential. The results, shown on Figure 7A, indicated a slight decrease in the proportion of cells with a high mitochondrial potential. However, no change in the value of this potential was observed upon treatment with hydroxyquinoline alone or with added copper. 

Lastly, we investigated the production of nitric oxide, which is one of the many differentiated functions of monocytes/macrophages. The results, shown on Figure 7B, indicated an increase in the basal production of nitric oxide, both by hydroxyquinoline alone and—even more strongly—with added copper. However, when lipopolysaccharide was also added to stimulate the cells and mimic a bacterial infection, the treatment with hydroxyquinoline alone showed no effect while the treatment with the copper complex induce a slight decrease in nitric oxide production. 

## 4. Discussion

With the increase in sensitivity of mass spectrometers, multi-identification of several proteins in a single two-dimensional gel spot has become increasingly frequent; this has been perceived as a problem by some authors [75] but not others [76]. It has been demonstrated that in most cases, the different proteins contained in a single spot are present in very different amounts, and that the variations detected via image analysis of 2D gels impact the most abundant protein in the spot [77]. As expected, the severity of the multi-identification issue increases with protein loads [78], both because of the increased diffusional spreading of proteins at high loads and because of the streaking induced by gel overloading. However, even in the harshest possible conditions, i.e., the coupling of a highly sensitive mass spectrometry with sub-milligram protein loads [64], the list of proteins identified in a single spot lengthens, but the first candidate usually accounts for more than 70% of the total mass spectrometry signal.

On the basis of these data, we decided to report only “winner takes all” identifications, but only in cases where this situation is most likely to occur, i.e., when the first candidate accounts for more than twice the number of unique peptides or spectra than the second candidate. A post-hoc validation of this strategy in the case of this study is represented by the fact that the proteins found as modulated belong to well identified pathways and are not randomly distributed. 

A good example of this is represented by the numerous proteins identified in the proteasome/ubiquitin pathway, known to be a target of the copper complexes [6,20,22,26]. With the exception of the immunoproteasome subunit psb8, all modulated proteasome subunits detected in the proteomic screen are decreased upon treatment with the copper complex, suggesting a destabilization of the proteasome itself. This effect has been shown to be copper-dependent [24], and coupling the data on the abundances of the proteasome subunits to our data on ion transport mediated by hydroxyquinoline in the absence of added copper further corroborated this finding. 

Indeed, beside the effects of the copper complexes, hydroxyquinoline derivatives by themselves have been shown to impact important biochemical processes such as the histone deacetylase pathway [79], the mTOR [80] or autophagy [81] pathways, or even the proteasome activity [45]. Our proteomic screen did not detect important effects of hydroxyquinoline alone. However, the effects reported to date have been observed only with higher doses (>10 µM) than the one used here (4 µM). 

In addition to proteasome inhibition, other effects such as redox effects have been reported for copper complexes [29,30]. Here again, the proteomic screen corroborated this hypothesis, with a decrease in the abundances of two abundant cytosolic peroxiredoxins (prx2 and prx6) and in a thioredoxin-like protein (txnl1) upon treatment with the copper complexes. 

These two examples suggest that a proteomic screen could be a powerful tool for the detection of other targets of the copper complexes, in a strategy similar to the one used for gold complexes [44]. Indeed, proteomics suggested that the level of reduced glutathione could be impacted by the copper complexes, which was confirmed by targeted experiments. This result is in line with what we previously showed for copper nanoparticles [65].

Another pathway that we found modulated by the copper treatment was the actin cytoskeleton, and here again our targeted experiments on cell shape and on phagocytosis confirmed the impact of the copper complexes on this pathway. This finding may be of interest in anticancer strategies, as the actin cytoskeleton is key to cell shape and motility, i.e., parameters that are important in the metastasis phenomenon. 

Regarding the mitochondria, whose proteins are well represented in the proteomic screen, we tested the mitochondrial potential. While a small decrease in the proportion of cells with a high mitochondrial potential was noted after treatment with the copper complex, corresponding to the slight increase in mortality induced by this treatment, the value of the mitochondrial potential did not change appreciably upon treatment by 8-hydroxyquinoline or to the 8-hydroxyquinoline-copper complex. This may mean that the changes observed in the mitochondrial proteins are sufficient to maintain the mitochondrial potential to a normal value.

Lastly, we investigated one differentiated function of monocytes/macrophages, namely the production of nitric oxide, either alone or after stimulation with lipopolysaccharide. Interestingly, both 8-hydroxyquinoline and its copper complex were able to induce by themselves a significant increase in the production of nitric oxide, a fact that may be related to the reported release of TNF alpha by clioquinol, a hydroxyquinoline derivative [82]. Regarding the nitric oxide release after stimulation with lipopolysaccharide, we observed a decrease upon treatment with the copper complex, in line with what we observed with copper oxide nanoparticles [83]. 

## 5. Conclusions

In conclusion, the main output of the proteomic study of the effects of the 8-hydroxyquinoline-copper complex on leukemic cells is the demonstration that beyond its well-documented effects on the proteasome, it also has multi-target effects on cells, including the oxidative stress response and the cytoskeleton. On the negative side, such a pleiotropic effect may increase the toxicity of the drug toward normal, non-cancerous cells. On the positive side, a pleiotropic effect strongly decreases the probability of emergence of resistant cells, and thus, may be highly beneficial as a cancer treatment. In line with this observation, metallodrugs, including copper complexes, are among the rare drugs which are able to target cancer stem cells [84], thereby representing a very important actor in the emergence of resistance and, therefore, in cancer relapses. Moreover, copper appears to have some selectivity against cancer cells [48,49]. Thus, the multipotency of metal complexes clearly deserves further research. 

## Figures and Tables

**Table 1:** Ion levels in cells.

Condition	Control	OHQ	Qcu
Zn	263 ± 81	257 ± 23	280 ± 79
Cu	16 ± 17	13 ± 10	1076 ± 258

The ion concentrations are expressed in ng ion/mg cellular protein. OHQ: treatment with 4 µM 8-hydroxyquinoline. Qcu: treatment with 4 µM 8-hydroxyquinoline +2 µM copper sulfate.

## Supplementary Materials

The following are available online at https://www.mdpi.com/2227-7382/7/2/26/s1, Figure S1:Raw gel images for the control cells, Figure S2: Raw gel images for the cells treated with the copper complex, Figure S3: Raw gel images for the cells treated with hydroxyquinoline alone, Table S1: Quantitative data from computerized gel analysis, Table S2: Protein identification data by mass spectrometry, Table S3: Expected false discovery rates for the proteins modulated by the hydroxyquinoline-copper complex, Table S4: Expected false discovery rates for the proteins modulated by hydroxyquinoline alone, Table S5 Modulated pathways highlighted by the DAVID annotation tool. Cells treated with hydroxyquinoline copper complex, Table S6: Modulated pathways highlighted by the DAVID annotation tool. Cells treated with hydroxyquinoline alone.
