# Supplementary figures and images for: A Proteomic View of Cellular Responses to Anticancer Quinoline-Copper Complexes
Source: Proteomes. 2019 Jun 24;7(2):26. doi: 10.3390/proteomes7020026 (PMC6630412; doi:10.3390/proteomes7020026)

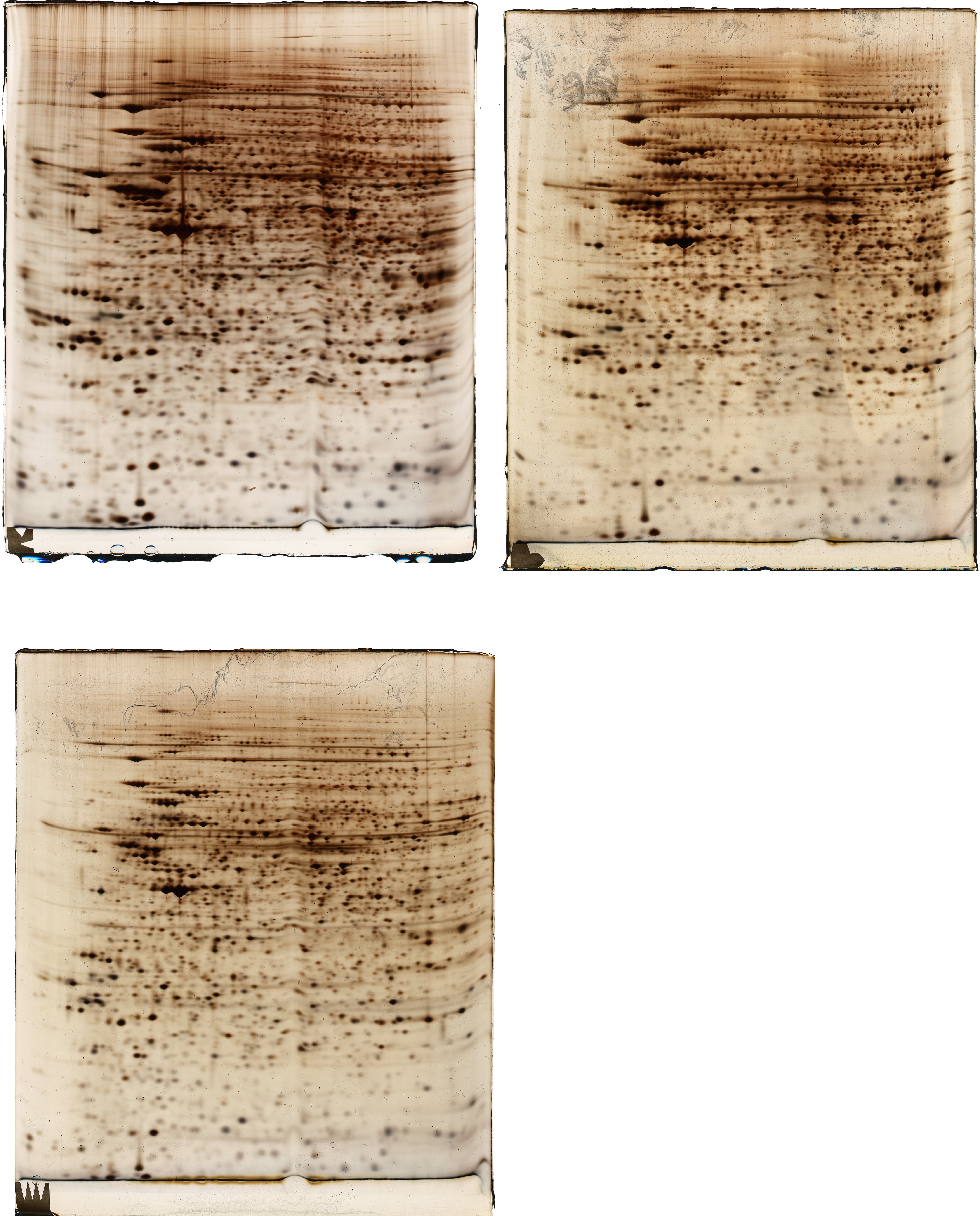

Supplement: Supplementary file 1 [file proteomes-07-00026-s001.zip › Figure S1.jpg]

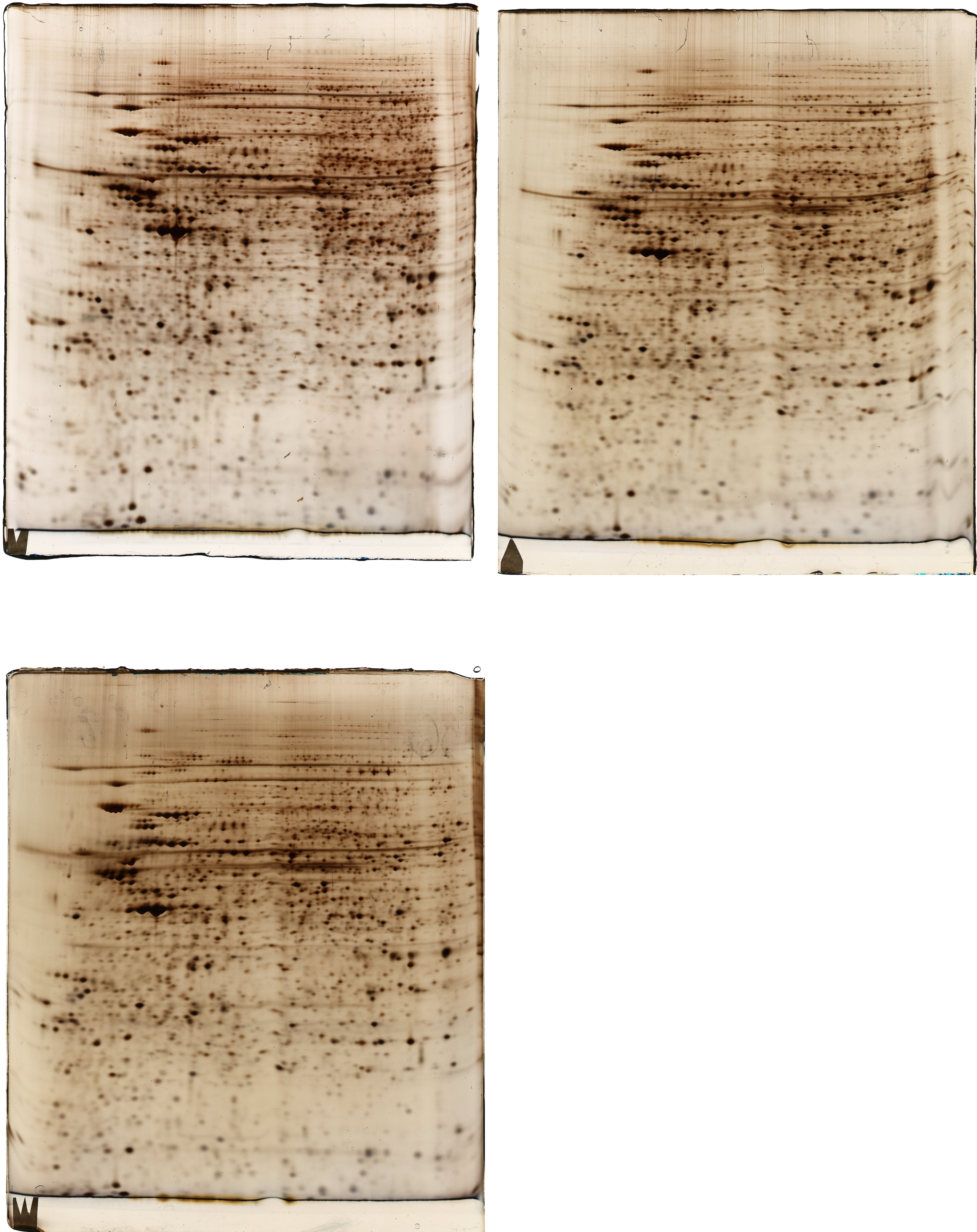

Supplement: Supplementary file 1 [file proteomes-07-00026-s001.zip › Figure S2.jpg]

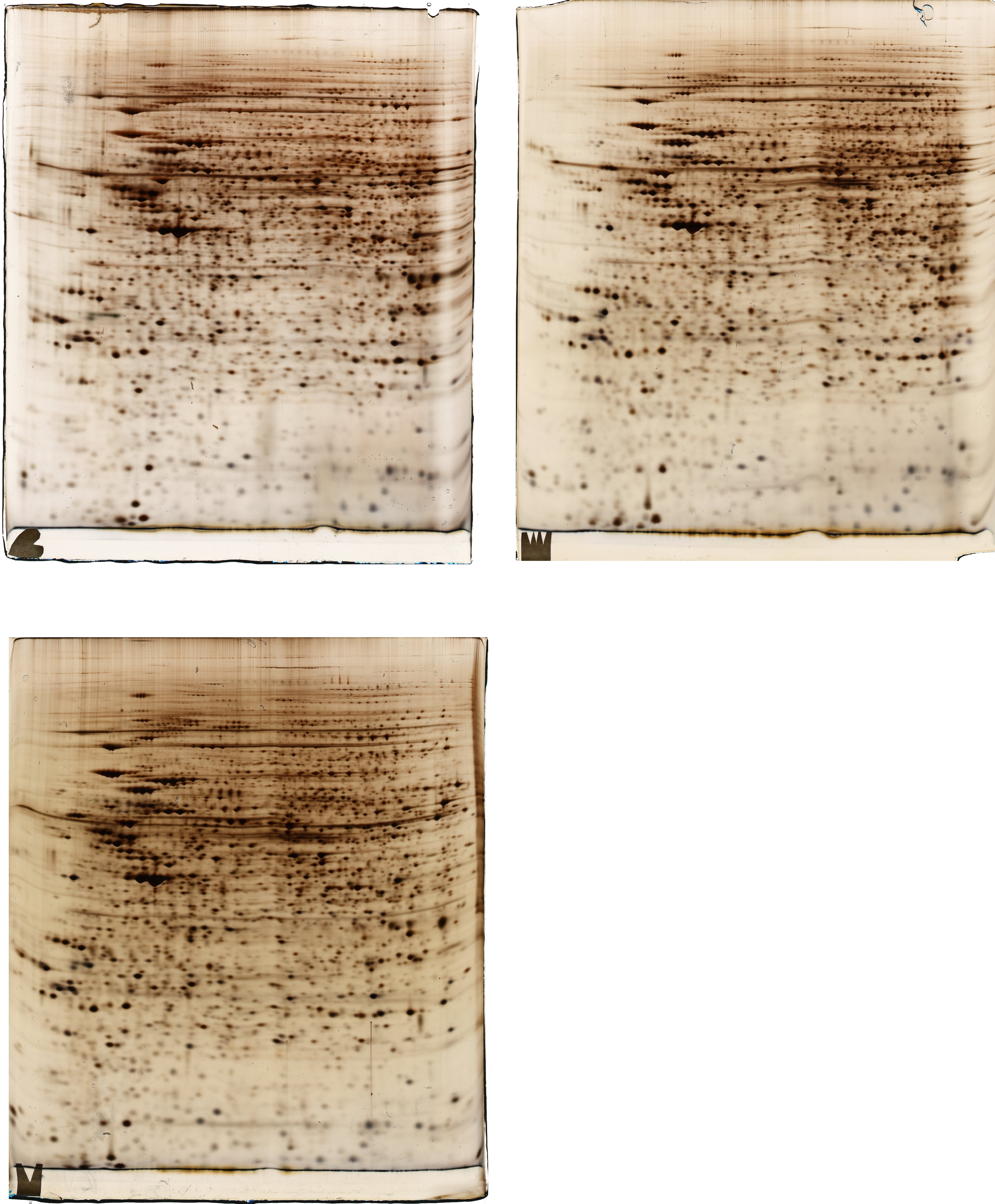

Supplement: Supplementary file 1 [file proteomes-07-00026-s001.zip › Figure S3.jpg]
